# Supplementary figures and images for: Inositol-Requiring Enzyme 1 pathway and autophagy drive sequential response of endothelial cells to febrile range hyperthermia
Source: PLoS One. 2025 May 7;20(5):e0315119. doi: 10.1371/journal.pone.0315119 (PMC12057933; doi:10.1371/journal.pone.0315119)

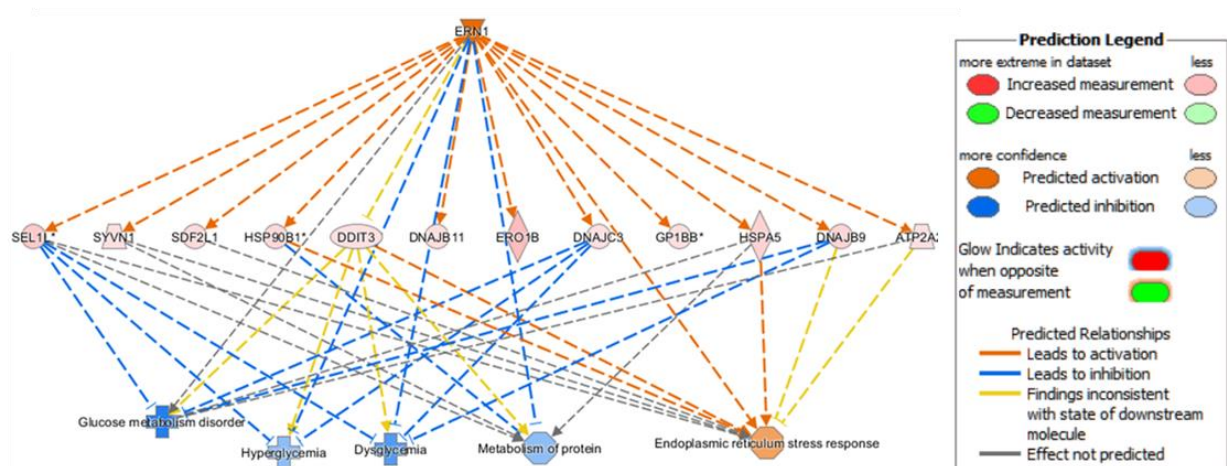

**Supporting Figure 1.** Top1 putative upstream regulator ERN1 (IRE1 $\alpha$ ) and its known targets.

Supplement: S1 Fig — . (PDF) [file pone.0315119.s001.pdf]

**Fig 4A**

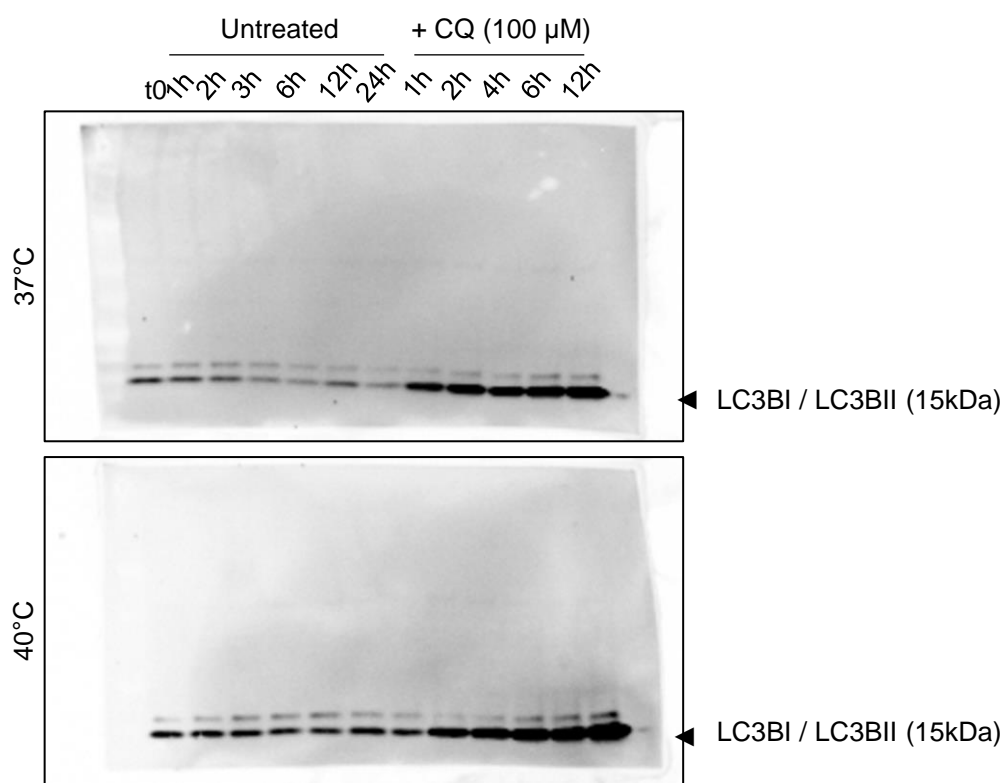

Fig 4B

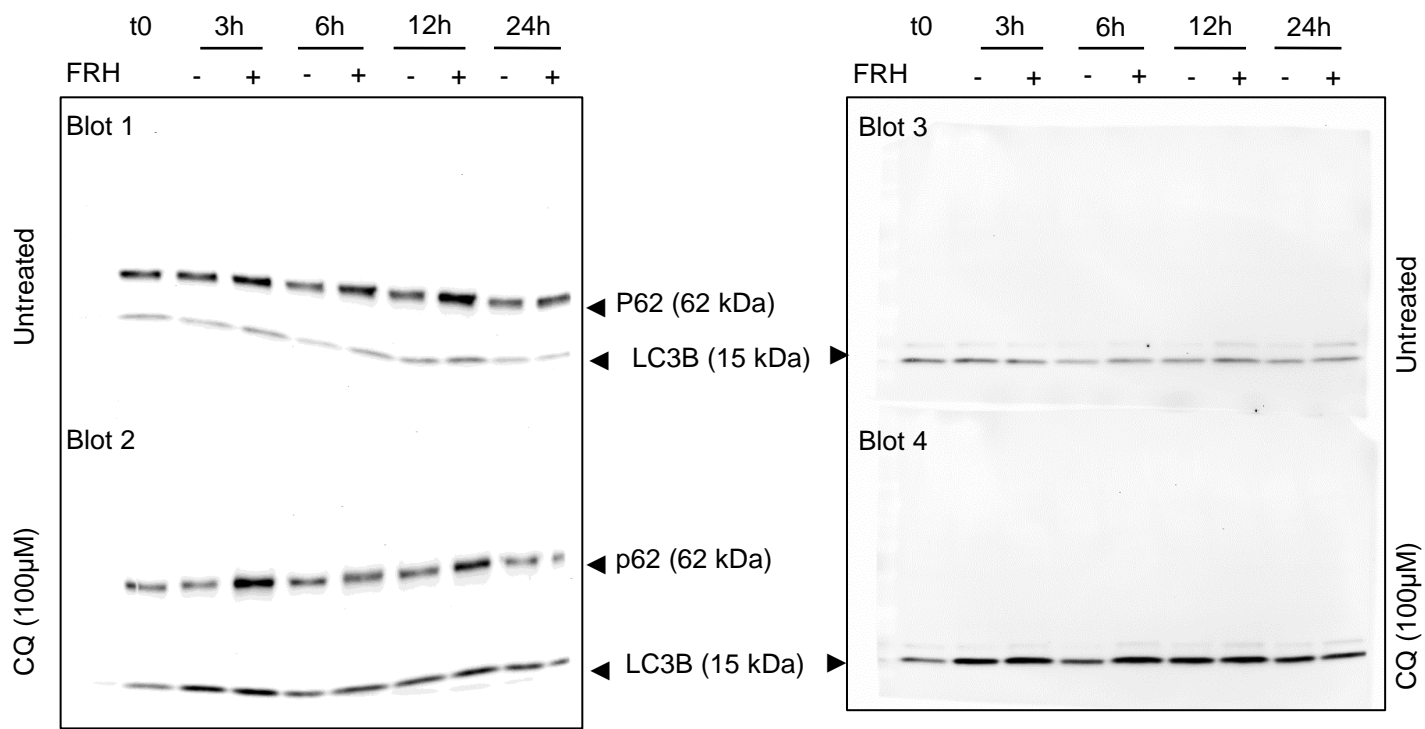

Fig 4B

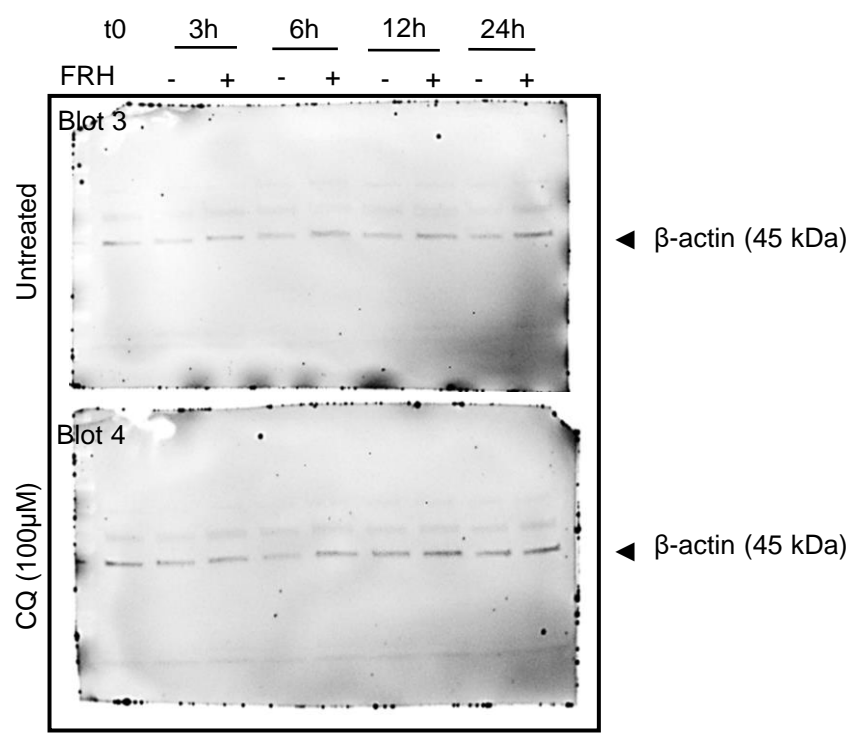

Fig 5D

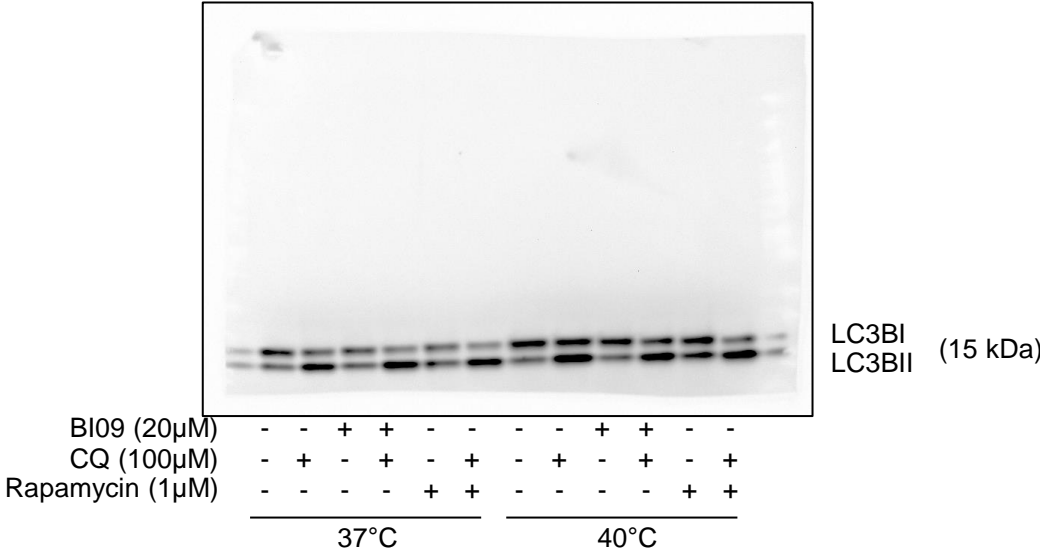

Fig 5F

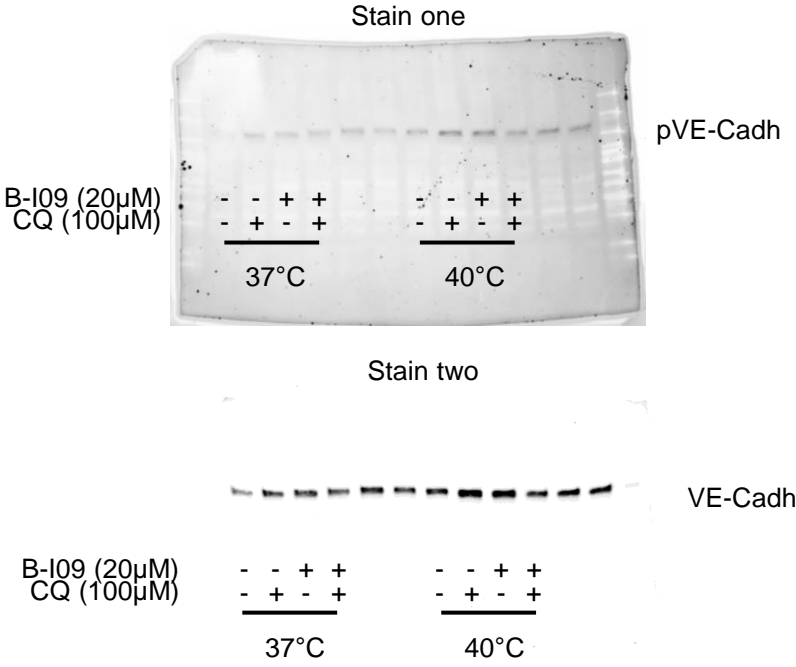

Supplement: S1 Data. Supporting information — (PDF) [file pone.0315119.s003.pdf]
