# Supplementary material for: Inositol-Requiring Enzyme 1 pathway and autophagy drive sequential response of endothelial cells to febrile range hyperthermia
Source: PLoS One. 2025 May 7;20(5):e0315119. doi: 10.1371/journal.pone.0315119 (PMC12057933; doi:10.1371/journal.pone.0315119)
Supplement: S2 Fig — A. Effect of B-I09 (20 μM) on sXBP1 and uXBP1 mRNA levels after 2h at 37°C or 40°C. B. Effect of B-I09 on the mRNA levels of DNAJB9 and HSP90B1. C. Level of phosphorylation of EiF2α in HCAECs subjected or not to FRH, assessed by Wes Simple Western System, and quantified (right). (PDF) [file pone.0315119.s002.pdf]

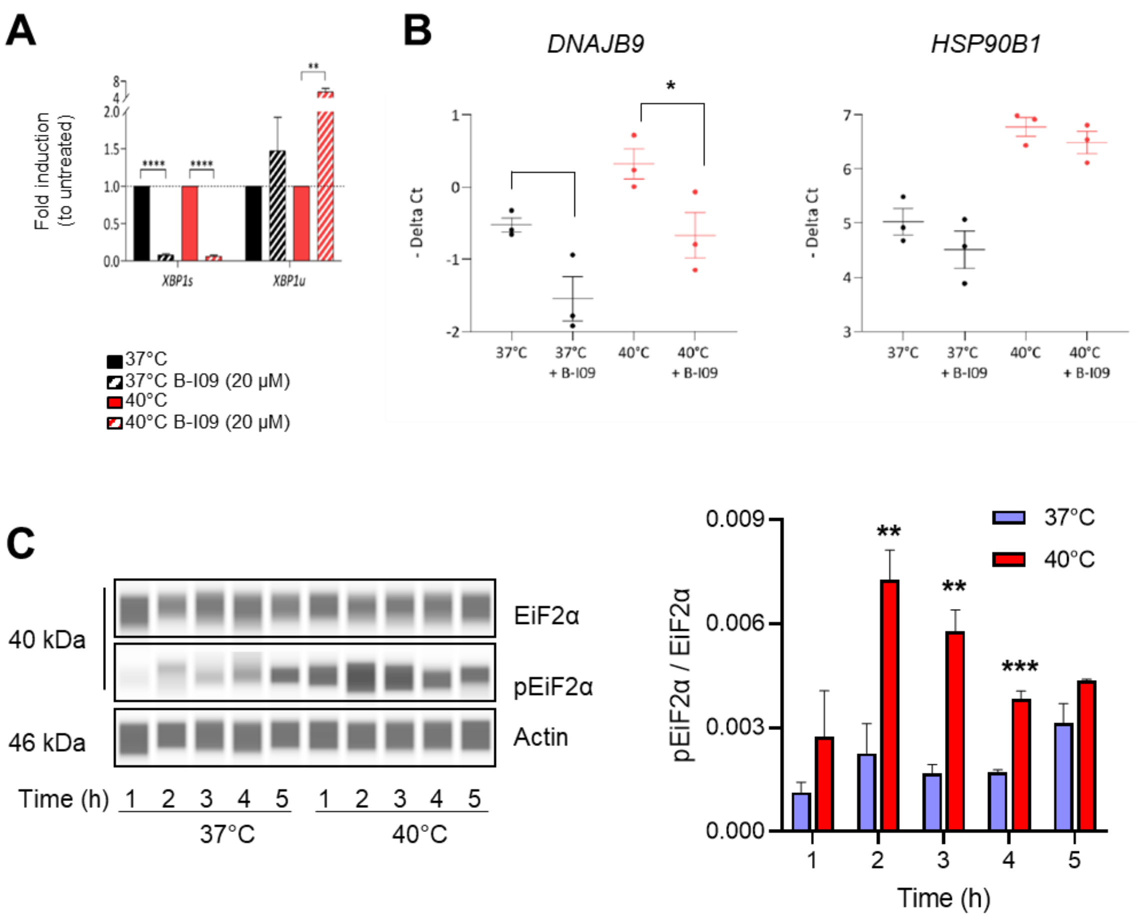

Supporting Figure 2. Effect of FRH on the unfolded protein response in HCAECs.

A. Effect of B-I09 (20 µM) on sXBP1 and uXBP1 mRNA levels after 2h at 37°C or 40°C. **B.** Effect of B-I09 on the mRNA levels of *DNAJB9* and *HSP90B1*. **C.** Level of phosphorylation of EiF2α in HCAECs subjected or not to FRH, assessed by Wes Simple Western System, and quantified (right).
